# Supplementary material for: Comparative Analysis of In-House RT-qPCR Detection of SARS-CoV-2 for Resource-Constrained Settings
Source: Diagnostics (Basel). 2022 Nov 21;12(11):2883. doi: 10.3390/diagnostics12112883 (PMC9689939; doi:10.3390/diagnostics12112883)
Supplement: Supplementary file 1 [file diagnostics-12-02883-s001.zip › diagnostics-1922684-supplementary.pdf]

Supplementary information

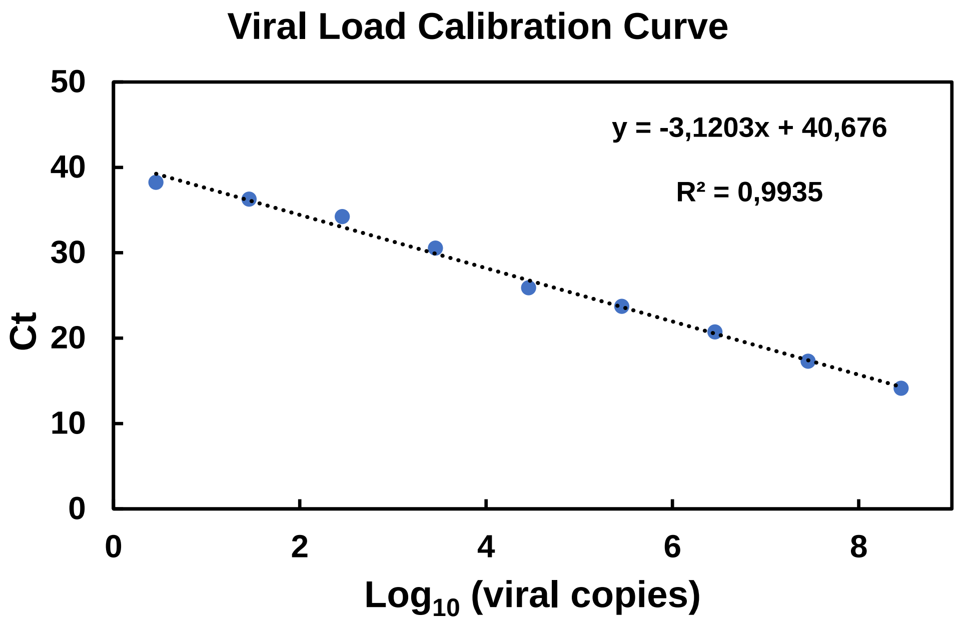

Figure S1. Calibration curve of the relationship between the number of viral copies (SARS-CoV-2) and Ct value from a diagnostic RT-qPCR. The calculation of the calibration line from the serial dilution of the 3180pb pUC series plasmid containing specific sequences of the viral E and RdRp genes detected by RT-qPCR related to the Ct value.

Table S1. Viral copy number ratio in each RNA dilution factor for samples positive for SARS-CoV-2.

Number of viral copies / μl of RNA	Dilution
**33863040.84**	1
**3386304.08**	1×10^{-1}
**338630.4**	1×10^{-2}
**33863.04**	1×10^{-3}
**3386.3**	1×10^{-4}
**338.64**	1×10^{-5}
**33.86**	1×10^{-6}
**3.38**	1×10^{-7}
**0.34**	1×10^{-8}

Table S2. Ct readings of SARS-CoV-2 detection tests by RT-qPCR using the GeneFinder™ triplex and duplex COVID-19 Plus kits.

GeneFinder™ COVID-19 Plus Kit						RT-qPCR Triplex				RT-qPCR Duplex^E			RT-qPCR Duplex^RdRp		
Gene	**IC**	**RdRp**	**E**	**N**		**IC**	**E**	**RdRp**		**IC**	**E**		**IC**	**RdRp**	
**Fluorophore**	Cy5 - control	FAM	Texas Red	VIC	Outcome	HEX - Control	Cal Red 610	FAM	Outcome	HEX - Control	Cal Red 610	Outcome	HEX - Control	FAM	Outcome
**Ct**	27,04	13,96	13,67	13,31	+	36,53	14,03	18,17	+	33,71	15,15	+	25,09	19,31	+
